# A Case of CDKL5 Deficiency Due to an X Chromosome Pericentric Inversion: Delineation of Structural Rearrangements as an Overlooked Recurrent Pathological Mechanism

**DOI:** 10.3390/ijms25136912

**Published:** 2024-06-24

**Authors:** Antonietta Lombardo, Lorenzo Sinibaldi, Silvia Genovese, Giorgia Catino, Valerio Mei, Daniele Pompili, Ester Sallicandro, Roberto Falasca, Maria Teresa Liambo, Maria Vittoria Faggiano, Maria Cristina Roberti, Maddalena Di Donato, Anna Vitelli, Serena Russo, Rosalinda Giannini, Alessia Micalizzi, Nicola Pietrafusa, Maria Cristina Digilio, Antonio Novelli, Lucia Fusco, Viola Alesi

**Affiliations:** 1 Laboratory of Medical Genetics, Translational Cytogenomics Research Unit, Bambino Gesù Children’s Hospital, IRCCS, 00165 Rome, Italy; 2 Medical Genetics Unit, IRCCS Bambino Gesù Children Hospital, 00165 Rome, Italy; 3 Medical Genetics Unit, San Pietro Fatebenefratelli Hospital, 00189 Rome, Italy; 4 Neurology, Epilepsy and Movement Disorders Unit, Bambino Gesù, IRCCS Children’s Hospital, 00165 Rome, Italy

**Keywords:** CDKL5 deficiency disorder (CDD), structural rearrangements, Xp22.13, *CDKL5*, epileptic encephalopathy, optical genome mapping, whole-genome sequencing

## Abstract

CDKL5 deficiency disorder (CDD) is an X-linked dominant epileptic encephalopathy, characterized by early-onset and drug-resistant seizures, psychomotor delay, and slight facial features. Genomic variants inactivating *CDKL5* or impairing its protein product kinase activity have been reported, making next-generation sequencing (NGS) and chromosomal microarray analysis (CMA) the standard diagnostic tests. We report a suspicious case of CDD in a female child who tested negative upon NGS and CMA and harbored an X chromosome de novo pericentric inversion. The use of recently developed genomic techniques (optical genome mapping and whole-genome sequencing) allowed us to finely characterize the breakpoints, with one of them interrupting *CDKL5* at intron 1. This is the fifth case of CDD reported in the scientific literature harboring a structural rearrangement on the X chromosome, providing evidence for the hypothesis that this type of anomaly can represent a recurrent pathogenic mechanism, whose frequency is likely underestimated, with it being overlooked by standard techniques. The identification of the molecular etiology of the disorder is extremely important in evaluating the pathological outcome and to better investigate the mechanisms associated with drug resistance, paving the way for the development of specific therapies. Karyotype and genomic techniques should be considered in all cases presenting with CDD without molecular confirmation.

## 1. Introduction

The *CDKL5* gene was first associated with a rare form of neonatal onset epileptic encephalopathy in 2004 [1,2], and in the years that followed, CDKL5 deficiency disorder (CDD) was identified as a diagnostically recognizable entity [3]. CDD is a rare and severe neurologic X-linked-dominant disorder, characterized by early-onset and drug-resistant seizures, developmental delay, intellectual disability, motor control and cortical visual impairment, absent speech, slight facial features, sleep disorder, and stereotypic movements [2,4,5,6]. The prevalence of the disorder is four times higher in females compared with males (4:1) [5], with a more severe clinical outcome usually being prevalent in the male population [7]. Infantile spasms are the initial seizure type documented in 23% of patients; however, as the disease progresses, epilepsy tends to be generalized or mixed in presentation, including spasms, tonic seizures, and tonic–clonic seizures. Seizures are usually brief, lasting <1 min but very frequent (>5 per day), and refractory to antiepileptic treatment and ketogenic diet [8]. Typically, interictal electroencephalographic activity shows high-amplitude delta waves with pseudo-periodic bursts of high-amplitude spikes, polyspikes, and spike waves that can either be generalized or localized in central, temporal, or temporo-occipital regions [8]. The neurological outcome of this condition is poor, and most patients are unable to walk or feed themselves independently. Communication is limited, based on elementary and non-verbal strategies. Of late, researchers have proposed minimal diagnostic criteria for CDD patients, which include the genetic finding of a pathogenic or likely pathogenic variant in *CDKL5*, in association with early-onset epilepsy and severe developmental delay [6]. *CDKL5* maps on the short arm of the X chromosome, at Xp22.13, consists of 18 exons (NM_ NM_001323289), and encodes a serine/threonine kinase. The gene plays an important role in axon growth regulation, dendritic morphogenesis, and synapsis function. *CDKL5* has several isoforms, with some of them presenting with ubiquitous expression and others predominantly reported in the brain [9]. The highest transcription levels are reported in glutamatergic and GABAergic neurons of forebrain structures (particularly in the hippocampus and cerebral cortex), with little or no expression in glial cells. The intracellular localization of the codified protein changes through development, presenting with an expression peak in early postnatal life when it is mostly located in the cytoplasm [9,10]. In vivo and in vitro studies show that decreased CDKL5 levels result in impaired dendritic branches, hampering synaptic formation and function [11,12]. Considering its pivotal role in brain structures, the severe neurological phenotype associated with CDD is not surprising. Even if the full function of CDKL5 has not been completely elucidated, its role in signaling and gene regulation through phosphorylation is known. Its phosphorylating activity is directed toward different substrates including the chromatin-associated protein MECP2, responsible for Rett syndrome (OMIM #312750) [1,13]. This finding suggests a common metabolic pathway for both proteins, which explains the partial phenotypic overlap of CDD with Rett syndrome despite being a distinct clinical entity [14].

Several pathogenic variants have been described in the literature, including deletions, nonsense, splicing, and missense variants [6]. Consistent with haploinsufficiency as the primary mechanism of disease, pathogenic missense variants have only been reported in the kinase domain, splice sites, and C-terminus regulatory domain, likely reducing kinase activity [14,15]. Individuals with *CDKL5* duplications have been described in the literature, showing a different phenotype, characterized by variable penetrance of macrocephaly and learning disability without seizures [16]. Only four cases of structural rearrangements have been reported in the scientific literature thus far [17,18,19].

Herein, we report a 3-year-old girl presenting with an epileptic encephalopathy, highly suggestive of CDD, and harboring a pericentric de novo inversion on chromosome X, disrupting the *CDKL5* coding sequence. We propose structural rearrangements as a possible recurrent mechanism associated with CDD.

## 2. Case Report

**Figure 1:**
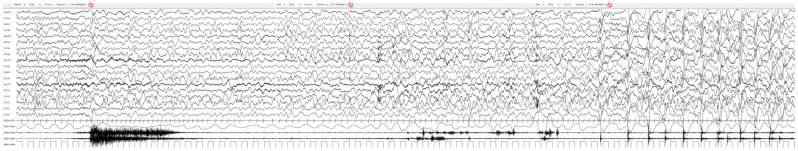
A brief bilateral tonic seizure, with a diffuse EEG counterpart, is followed by a cluster of periodic myoclonias synchronized with periodic spike-wave complexes on the EEG.

**Figure 2:**
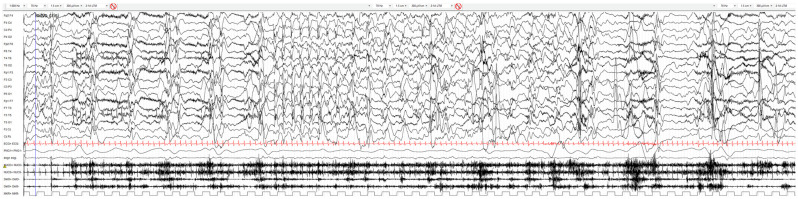
The seizure begins with a single myoclonus associated with a diffuse spike, followed by epileptic spasms associated with periodic high-amplitude slow waves and then again by myoclonias with a spike-wave EEG pattern. Myoclonias and spasms are interspersed.

Herein, we report on a young girl evaluated for the first time at three years of age at the Neurological Department of Bambino Gesù Children’s Hospital in Rome, Italy, showing a severe epileptic encephalopathy. She is the only child of an unrelated, healthy couple. She was born at 39 weeks of gestation by spontaneous delivery after an uneventful pregnancy. Her weight at birth was 3.030 g (25° centile), her length was 51 cm (50° centile), and her occipital–frontal circumference was 33 cm (10th–25th percentile). Her Apgar score was 8 and 9 at the first and fifth minutes of birth, respectively. The perinatal period was reported as normal. The girl’s first seizure occurred at three months of age. The patient was admitted to the hospital because of poor visual engagement, delayed head control, and episodes of predominantly leftward eye deviation lasting roughly one minute, associated with poor responsiveness, resolving spontaneously. Upon admission, a delay in motor and developmental milestones was noted as she was not able to control her head or fixate her gaze. The results of brain MRI with contrast medium showed no anomalies. Her metabolic screening results, including pterines on the spot, dihydrobiopterin reductase (DHPR) activity, aminoacidic profile, and biogenic amines in cerebrospinal fluid, were found to be normal. The results of her wake and sleep electroencephalogram (EEG) showed polymorphic and asymmetric theta activity due to the presence of higher voltage activity in the posterior regions of the right hemisphere, along with slow spike-wave type anomalies. During the recording, the emergence of fast activity in the fronto-temporo-occipital regions on the left side was recorded, preceded by a slow wave of high voltage and tonic contraction of the upper limbs. Therapy with carbamazepine was started. At the age of 4 months, the girl was admitted to our hospital. Developmental delay was confirmed. Long-term video/EEG monitoring recorded seizures appearing as tonic seizures followed by bursts of periodic myoclonias (Figure 1). The EEG pattern promptly suggested the etiological diagnosis of a CDKL5 encephalopathy, as is typical of this type of developmental disorder [20]. Subsequently, other seizures occurred in the following months, including focal seizures and hypermotor seizures interspersed with tonic seizures, also described in CDKL5 encephalopathy and suggestive of this clinical entity. Epileptic spasms appeared at the age of 7 months, persisting with a fluctuating course, often interspersed in a single seizure with myoclonias (Figure 2). Because of the emergence of maximum myoclonic jerks, clonazepam therapy was initiated, leading to a slight reduction in episode frequency. Subsequently, the patient’s epilepsy became refractory to both first- and second-line antiepileptic drugs and the patient underwent multiple hospitalizations. Control EEG recorded globally pathological, disorganized, and unstructured brain activity in wakefulness and discontinuous activity during sleep. The recording exhibited bilateral multifocal slow and epileptiform abnormalities, as well as epileptic myoclonus and infraclinical sequences or minimal clinical components, with widespread expression. Clinical genetic examination revealed absent visual connection, epicanthus, a saddled nasal root, a long and deep philtrum, downward corners of the mouth, and partial control of the head. The patient presented with scoliosis and bilateral hip subluxation. The patient’s occipital–frontal circumference was 48 cm (15th-50th percentile). She presented with severe psychomotor delay, generalized hypotonia with mobilization beyond the physiological joint range, aposturality, severe intellectual disability, and absent speech. The clinical findings, along with the EEG pattern, were suggestive of a *CDKL5*-related epileptic encephalopathy. 

## 3. Results

The patient tested negative during the NGS analysis of genes related to epilepsy. A paternally inherited heterozygous missense VUS variant (a variant of unknown significance: NM_000833 (GRIN2A): c.[2551G>C];[=] p.([Gly851Arg];[=]) in the *GRIN2A* gene was detected and considered not to be associated with the clinical phenotype. 

**Figure 3:**
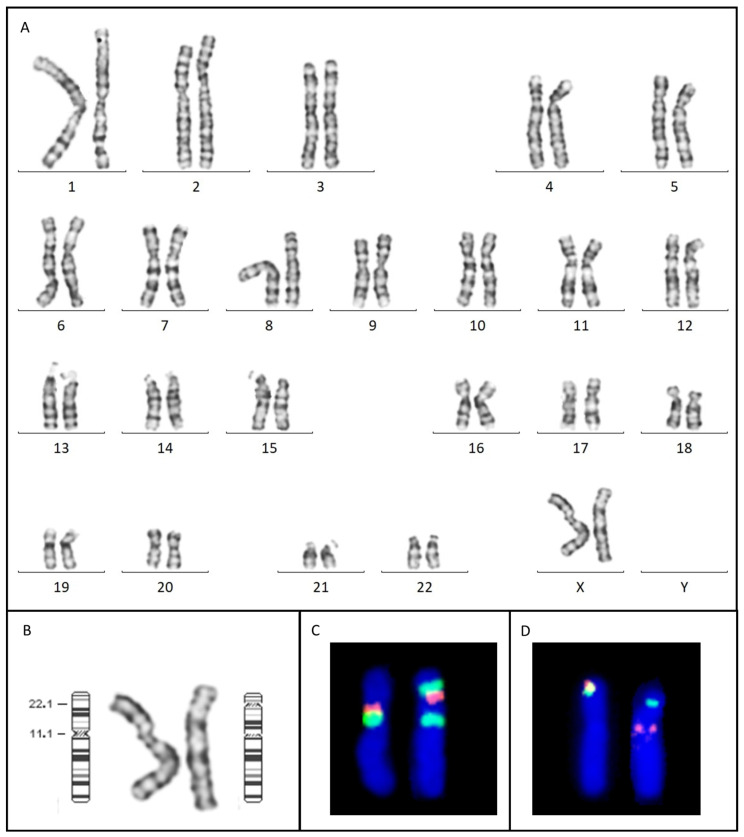
(**A**) G-banding karyotype: female euploid karyotype, harboring a pericentric inversion on the X chromosome [46,X,inv(X)(p22.12q11)]. (**B**) Ideograms of the two X chromosomes showing the inversion breakpoints. (**C**) FISH assay using Xp11.1q11.1 Alpha Satellite (green) and NYX (orange, Xp11.4) probes characterizing the proximal breakpoint of the inversion. The two green fluorescent signals on the rearranged X chromosome (right) prove that one of the breakpoints involved the pericentromeric region. The NYX orange signal moves distally, between the two green signals. (**D**) FISH assay using a custom-designed oligonucleotide probe characterizing the distal breakpoint of the inversion, involving *CDKL5*. The Xp22.13 (green)/*CDKL5* (orange) probe signals, normally joining at the extremity of the X chromosome short arm, split on the two arms of the rearranged chromosome.

The results of karyotype analysis showed a female karyotype with a pericentric inversion of an X chromosome, with breakpoints at Xp22.12 and Xq11 [46,X,inv(X)(p22.12q11)dn] (fig3.A). Cytogenetic analysis performed on both parents confirmed that the identified structural rearrangement occurred de novo. The patient tested negative during CMA analysis, showing that the inversion was not associated with genomic material loss.

The inversion was characterized using FISH analysis, which excluded the involvement of other chromosomes in the structural rearrangement (Figure 3B,C). 

The cytogenetic results, associated with the clinical presentation and EEG, highly concordant with CDD, pointed our attention toward *CDKL5*, which was considered likely involved in the structural rearrangement. In order to provide a better characterization of the inversion, optical genome mapping (OGM) and whole-genome sequencing (WGS) were performed.

**Figure 4:**
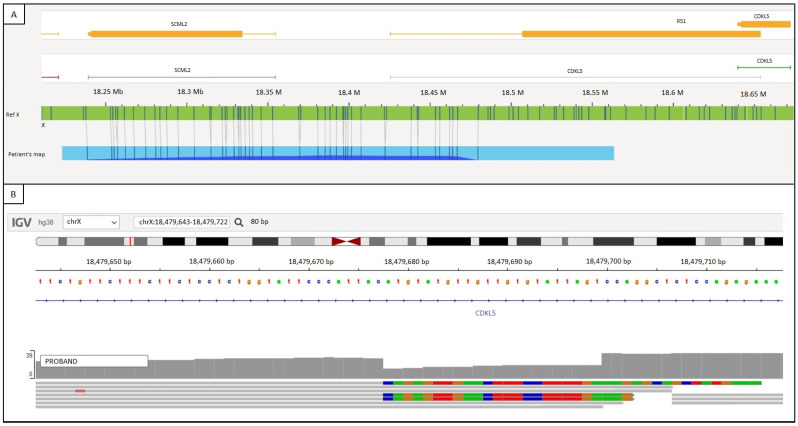
Genomic analysis of peripheral blood from the patient was performed, characterizing the inversion breakpoints at Xp22.13. (**A**) The patient’s optical map (blue bar) was aligned and compared with the reference optical map (green bar). Molecular labels are reported as vertical lines on both the patient’s and reference’s maps. The patient’s map only matches the reference’s map in the first gene portion, with the distal breakpoint of the inversion being in the pericentromeric region of Xq, where the high chromatin condensation does not allow for enzymatic labeling. (**B**) WGS data focusing on *CDKL5* (NM_001323289) show a misalignment at the intron 1 level (the alternative sequence is reported as colored segments, representing each nucleotide: green for A, blue for C, red for T, orange for G).

OGM software (Bionano Access v1.6) was unable to define the chromosomal structural rearrangement. However, when zooming in on the *CDKL5* region, anomalous maps were noted, showing the presence of aberrant molecules only matching the reference in the first gene portion (5’ regulatory sequence, exon 1, and part of intron 1) of *CDKL5*. The remaining part of the patient’s physical map presented no specific labels, hampering the software’s capacity to recognize the rearrangement (Figure 4A). Unsurprisingly, the distal breakpoint of the inversion was located in the pericentromeric region of the X chromosome long arm, where high chromatin condensation does not allow the enzyme to associate the fluorochrome to its specific DNA sequence target. 

WGS data were analyzed focusing on *CDKL5* (NM_001323289). A misalignment was visible at the intron 1 level but could not be better characterized because of the nonunique sequence of the alternative allele, presenting several matches within the genome, as expected for pericentromeric regions (Figure 4B). 

**Figure 5:**
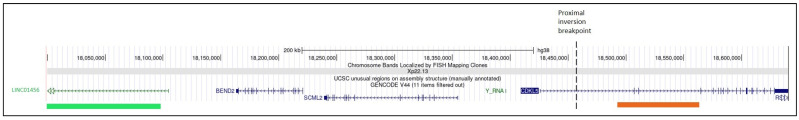
Custom Xp22.13/*CDKL5* FISH probe design. A red-labeled probe was designed on the *CDKL5* gene sequence (orange segment) while a green-labeled one was located about 589 kb upstream (green segment).

The *CDKL5* interruption was confirmed via FISH analysis using a custom locus-specific probe, specially designed to cover the regions located up- and downstream of the detected breakpoint (Figure 3D and Figure 5).

The results of the X chromosome inactivation test, on DNA extracted from the patient’s peripheral blood, showed a random inactivation pattern.

## 4. Discussion

We report on a young girl affected by a drug-resistant epileptic encephalopathy presenting with a clinical phenotype highly suggestive of CDD. The use of NGS and CMA analysis failed to detect a pathogenic variant involving the causative gene *CDKL5*, while the karyotype analysis results showed a de novo pericentric inversion of the X chromosome: 46,X,inv(X)(p22.12q11)dn. Pericentric inversions result from a 180° rotation of the chromosomal region included between two breakpoints, occurring on the two arms of the same chromosome [21]. Structural rearrangements are not a rare finding, as they are recorded in roughly 0.7% of the human population [22]. In particular, inversions present with an incidence ranging from 1–2/100 for autosomal chromosomes to 1/28,000–1/30,000 for sex chromosomes [23]. 

When not associated with the gain or loss of genomic material, structural rearrangements are often considered benign findings. However, they can result in pathological outcomes if gene expression is hampered by a physical interruption of a coding gene sequence, a chimeric or truncated gene product formation, or a functional expression domain alteration. Therefore, a patient harboring a balanced de novo rearrangement is considered to have a residual mortality risk of about 27% [24]. The fine characterization of the breakpoints is crucial in reaching a diagnosis, and this process has long been carried out via FISH analysis, with the use of locus-specific probes designed to consolidate the region of interest. However, this procedure is expensive to perform and time-consuming, and it is often not applicable in a diagnostic routine setting. For this reason, carriers of balanced structural rearrangements are usually given only a residual risk, without providing a precise molecular diagnosis. Emerging genomic techniques, such as OGM and WGS, are proving extremely useful in breakpoint characterization, as they provide a much higher resolution than cytogenetics and allow for a molecular diagnosis to be made [25,26]. However, the interpretation of the huge amounts of data produced by these platforms is challenging and requires the application of accurate analytical filters. Moreover, a direct and continuous interface with clinicians is necessary in order to address the analytical results correctly. 

Our patient tested negative in the genetic assays usually performed for epileptic encephalopathies (NGS and CMA), with the only positive result provided by standard karyotype analysis. However, the cytogenetic resolution did not allow for the proper characterization of the rearrangement to the point of identifying the exact localization of the breakpoints, and the genomic techniques used for this purpose (OGM and WGS) failed to determine the structural anomaly as well. The strong clinical suspicion of CDD led to a focus on *CDKL5*, allowing for locus-specific analysis. In evaluating the region with hindsight, the following peculiarities emerged: OGM showed aberrant maps, with them only matching the reference for the first portion of the gene, while WGS showed a mismatch at intron 1. For both techniques, accurate data interpretation was hampered by the physical features of the involved region, with this being the proximal breakpoint located within the pericentromeric chromosomal region, enriched with highly repetitive sequences. Therefore, clinical suspicion accuracy was of paramount importance since the diagnosis would be missed if made based on laboratory results alone, even if using the most innovative and recently developed techniques. 

Three unrelated female patients affected by CDD harboring chromosomal structural rearrangements involving *CDKL5* have been described in the scientific literature. They presented with a de novo balanced X-autosomal translocation, involving *CDKL5* at introns 10, 1, and 17 [17,18]. In particular, the first case reported by Kalsheuer et al. [17] showed frequent tonic–clonic seizures coupled with motor retardation and hypotonia at the age of 4.5 months and dyskinesias with short myoclonic-like movements occurring after the age of 7 years. Upon physical examination, she showed hypertelorism, a high nasal bridge, large ears, a low posterior hairline, and a simian crease. The patient died at the age of 17 years. In the second case reported by Kalsheuer et al. in [17], an EEG performed on a patient at the age of 2 months showed hypsarrhythmia, leading to a diagnosis of infantile spasms. The seizures evolved into tonic seizures not controlled by antiepileptic drugs and persisted until her death at 3 years of age [17]. In all the cases, the results of the X chromosome inactivation assay showed complete inactivation of the non-rearranged X chromosome, harboring the *CDKL5* wild-type allele, as expected in the case of X-autosomal translocation. In fact, the presence of an autosomal chromosome portion on the derivative X prevents it from inactivating, with autosomal material silencing likely having a strong impact on cell vitality. Moreover, the *CDKL5* transcription analysis, performed in two out of the three patients [17], showed the complete absence of the normal allele, demonstrating that *CDKL5* does not escape inactivation. In contrast, the results of the X inactivation test did not show a preferential pattern in our patient, indicating that neither *CDKL5* loss of function nor the chromosomal inversion are associated with cellular growth restriction, thus not leading to skewed inactivation of the affected X chromosome. The expression of a certain amount of wild-type protein product, consistent with random inactivation, did not prevent our patient from manifesting a clinical phenotype, as previously shown in the literature for cases harboring pathogenic single nucleotide variants [27]. The relationship between genetic variations and the CDD clinical spectrum has already been investigated by several authors, particularly concerning *CDKL5* point mutations [14,28]. However, there is still limited evidence regarding a clear genotype–phenotype correlation, and other factors, including somatic mosaicism and epigenetic mechanisms, have been implicated in phenotype modulation [29,30,31,32]. In this context, the inactivation of the X chromosome is reported as one of the most significant factors determining the severity of symptoms in the female population [7,33], and a distinction can be identified between the clinical implications of translocations and other types of chromosomal rearrangements. Similar to the observations noted in affected male patients, who did not express functional CDKL5 in any of their cells, the complete absence of CDKL5 in patients harboring X-autosomal translocation likely results in a more severe phenotype. Despite displaying a certain degree of clinical variability, CDD in males tends to present a more severe course and worse neurological outcome (marked by early-onset refractory epilepsy, profound general developmental delay, cortical visual impairment, compromised motor skills, and the absence of speech) [31,32,34]. While more than half of female patients [6] demonstrate motor development enabling them to sit or reach independent walking, males are often bedridden with intractable seizures [31,35]. Likewise, all three reported female patients harboring a balanced X-autosomal translocation involving *CDKL5* exhibited a severe phenotype, and two of them experienced an unfavorable outcome, leading to death within the second decade of life [17,36]. The above findings, coupled with the evidence of phenotypic divergence in familial *CDKL5-*positive cases, suggest a pivotal role of the X-inactivation pattern in determining the clinical picture [4,37].

A fourth patient was reported in the scientific literature harboring a mosaic structural micro-rearrangement involving *CDKL5*, detected via OGM [19]. The patient was male and presented with infantile spasms and tonic–clonic seizures at the age of one month. He showed refractory epilepsy, severe hypotonia, cortical visual impairment, and severe global developmental delay at the age of four years. Upon physical examination, coarse facial features, anteverted nares, and thick, prominent lips with tongue protrusion were noted. He also showed hirsutism, mild lower extremity hemihypertrophy, and small hands and feet. He carried an unbalanced inversion associated with a 90 kb microdeletion at one of the breakpoints, involving the regulation region and exons 1-3 of *CDKL5* [19]. Although it would probably have been possible to detect the microdeletion by means of a high-resolution microarray platform, leading to the final diagnosis of CDD, the small size of the inversion (only 10 kb in size) prevented its detection via karyotyping, overlooking the structural rearrangement as the etiological mechanism.

Our patient is the fifth patient described in the scientific literature harboring a structural variant inactivating *CDKL5*. The physical gene breakpoint is not the same in all five patients; therefore, a specific recombination hotspot is not distinguishable. However, the recurrence of an SR in the five different patients provides evidence for the hypothesis that this gene may be particularly prone to structural modification and recombination, possibly because of the presence of small high-homology sequences. Structural rearrangements may be a recurrent etiological mechanism in patients affected by CDD, and their incidence may be underestimated, with them being overlooked when standard diagnostic techniques are used. More studies are needed for a complete comprehension of these types of anomalies and to evaluate their frequency as a cause of disease onset. Cytogenetic and genomic analysis should be considered in patients with a clinical suspicion of CDD but who test negative when using standard techniques.

## 5. Materials and Methods

### 5.1. Cytogenetics and FISH Analysis

GTG-banding karyotype analysis was performed according to standard procedures for metaphases obtained from PHA-stimulated lymphocytes. Chromosome analysis of the patient and her parents was carried out at the 550-band level.

FISH analysis was performed to characterize the cytogenetic and OGM results. The probes used are listed below:

Using a whole painting X chromosome, a centromeric probe, and locus-specific probes mapping at Xp22.13, Xp22.12, Xp11.22, and Xp11.4:

- Whole X chromosome painting (ASI Applied Spectral Imaging, Carlsbad, CA, USA).
- Xp11.1q11.1 Alpha Satellite DNA (Aneuvysion Multicolor DNA probe kit, Vysis, Des Plaines, IL, USA).
- Custom-designed oligonucleotide FISH Probe, Agilent SureDesign production, mapping at [GRCh38]NYX (Xp11.4: 41442409-41542703), Xp22.12(Xp22.12:21374272-21474561), *CDKL5* (Xp22.13:18489962-18589141), and Xp22.13(Xp22.13:17999851-18099682).
- PR11-805H4 (Xp11.22) and 80N6 (Xp22.13): BAC probes selected from the University of California Santa Cruz (UCSC) http://genome.ucsc.edu genome browser (University of California, Santa Cruz, CA, accessed on 6 July 2023).

### 5.2. Clinical Exome Sequencing

A trio-based NGS epileptic encephalopathy in silico panel was performed on the genomic DNA by using the Twist Custom Panel (clinical exome—Twist Bioscience) according to the manufacturer’s protocol on a NovaSeq6000 platform (Illumina San Diego, CA, USA). Sequence data were aligned to the human genome build UCSC GRCh37/hg19. The Dragen Enrichment application of BaseSpace (Illumina, San Diego, CA, USA) and Geneyx Analysis software v5.17 (LifeMap Sciences, Inc. Alameda, CA, USA) were used for variant calling and variant annotation, respectively. All variants were checked in the public databases dbSNP (http://www.ncbi.nlm.nih.gov/projects/SNP, accessed on 6 July 2023) and gnomAD (http://gnomad.broadinstitute.org/, accessed on 6 July 2023) and filtered for an allele frequency < 1% in gnomAD. The variants were interpreted using VarSome [38] and categorized in accordance with American College of Medical Genetics (ACMG) recommendations [39]. The variants were examined for a minimum depth coverage of 30× and Qscore (minimum threshold of 30) and visualized using the Integrative Genome Viewer (IGV) [40].

### 5.3. Chromosomal Microarray Analysis (CMA)

Genomic DNA was extracted from the patient’s peripheral blood with Qiagen columns (QIAamp DNA minikit; Qiagen, Hilden, Germany). Chromosomal microarray analysis (CMA) was performed using Infinium CytoSNP-850K BeadChip (Illumina, San Diego, CA, USA), according to the manufacturer’s protocol. Array scanning data were generated on the Illumina NextSeq 550 system, and the results were analyzed using Bluefuse Multi 4.4 software.

### 5.4. Optical Genome Mapping (OGM) and Structural Variant Calling 

Optical genome mapping is a new non-sequencing imaging tool that provides high-resolution information about the presence of copy numbers and structural variants across the entire genome. The technology is based on the isolation of ultra-high molecular weight DNA, which is uniquely labeled, directly imaged, and used for building an accurate physical genome map. Comparative analysis of the label patterns over long contiguous reads across the entire genome reveals the occurrence of both copy number variants and structural variants.

A fresh blood aliquot, collected in EDTA tubes, was stored at −80 °C immediately after sampling. Ultra-high molecular weight (UHMW) DNA was extracted according to the manufacturer’s instructions (SP Frozen Human Blood DNA Isolation Protocol, Bionano Genomics, San Diego, CA, USA) and enzymatically labeled using the DLE-1 Enzyme (Bionano Prep Direct Label and Stain Protocol). Labeled DNA was loaded on Saphyr chips and scanned using the Saphyr instrument (Bionano genomics, San Diego USA). The Saphyr chips were run to reach a minimum yield of 320 Gbp corresponding to 100× effective coverage. The de novo assembly and variant annotation pipeline were executed on Bionano Solve software V3.6 using the Human Genome Reference Consortium GRCh38 assembly as a reference for structural variant detection. Reporting and direct visualization of the structural variants were performed on Bionano Access V1.6.

### 5.5. Whole-Genome Sequencing (WGS)

WGS was performed on genomic DNA in order to provide fine characterization of the translocation events. Library preparation was carried out according to the manufacturer’s protocol using DNA PCR-Free Library Prep (Illumina) and sequenced on a NovaSeq6000 (Illumina) platform. The obtained NGS (next-generation sequencing) assay presented a mean coverage of 35×, with Q30 bases around 87%. TruSight Software Suite v2.6.3 (Illumina), the integrated DRAGEN platform, and IGV software were used for alignment, variant calling, and breakpoint data visualization. Sequencing data were aligned to the hg38 human reference genome.

### 5.6. X Chromosome Inactivation Pattern

X chromosome inactivation was evaluated on DNA extracted from the patient’s peripheral blood. The methylation pattern was assessed on a polymorphic repeated locus in the human androgen receptor (AR) gene at Xq12, containing an HpaII methylation-sensitive restriction site, as previously described [41]. Hpa II-digested genomic DNA was amplified using a forward FAM-tagged primer (forward sequence: [6FAM]GCTGTGAAGGTTGCTGTTCCTCAC; reverse primer: TCCGAATCTGTTCCAGAGCGTGC). The PCR products were analyzed on an ABI PRISM® 3100 Genetic analyzer (Applied Biosystem, Waltham, MA, USA), using GeneScan™ 500 ROX™ (Applied Biosystems) as an internal lane size standard. GeneMapperTM Software v4.0 (Applied Biosystems) was used to calculate the position of the peaks and to determine area intensity. X-inactivation was undertaken at the HUMAR locus as previously described [42]. X-inactivation ratios were calculated as the areas under the curve (AUC) for each allele [25]. XCI ratios equal to or less than 80:20 were considered “random” patterns, while ratios greater than 80:20 were considered “skewed” patterns, according to published criteria [41,43,44,45].

## 6. Conclusions

In the late 1970s, most cases of epilepsy were defined as idiopathic. Over the last 50 years, many advances have been made, and to date, cases of epilepsy of unknown cause represent only a small portion of cases thanks to the identification of several immunological, structural, and genetic causes of the condition. CDD is a rare X-linked dominant genetic condition associated with severe and drug-resistant epileptic encephalopathies caused by the loss of function of the *CDKL5* gene. Even if point mutations are reported as the main cause of gene inactivation, in our patient, as well as in four others described in the scientific literature, a structural rearrangement interrupting the *CDKL5* coding sequence has been shown. SRs are challenging to detect because they are overlooked by standard laboratory techniques, and therefore, their frequency is likely underestimated. Knowing the genetic basis of the disease is of paramount importance to correlate the genetic defect with the severity and progression of the pathology. For example, even if the clinical outcome is usually less severe in females than in males, the presence of an X-autosome translocation in a female patient can result in a poorer prognosis because of the complete inactivation of the non-rearranged X chromosome harboring the wild-type allele. Furthermore, studying the molecular causes underlying the onset of the pathology will allow for a better understanding of the mechanisms associated with drug resistance, paving the way for the development of new, specific therapies.

## Data Availability

NGS, CMA, and OGM data are available from the authors upon request.

## References

[1] Tao J., Van Esch H., Hagedorn-Greiwe M., Hoffmann K., Moser B., Raynaud M., Sperner J., Fryns J.P., Schwinger E., Gécz J. (2004). Mutations in the X-linked Cyclin-Dependent Kinase-Like 5 (CDKL5/STK9) Gene are Associated with Severe Neurodevelopmental Retardation. Am. J. Hum. Genet..

[2] Leonard H., Downs J., Benke T.A., Swanson L., Olson H., Demarest S. (2022). CDKL5 Deficiency Disorder: Clinical Features, Diagnosis, and Management. Lancet Neurol..

[3] Demarest S.T., Olson H.E., Moss A., Pestana-Knight E., Zhang X., Parikh S., Swanson L.C., Riley K.D., Bazin G.A., Angione K. (2019). CDKL5 Deficiency Disorder: Relationship between Genotype, Epilepsy, Cortical Visual Impairment, and Development. Epilepsia.

[4] Weaving L.S., Christodoulou J., Williamson S.L., Friend K.L., McKenzie O.L., Archer H., Evans J., Clarke A., Pelka G.J., Tam P.P. (2004). Mutations of CDKL5 Cause a Severe Neurodevelopmental Disorder with Infantile Spasms and Mental Retardation. Am. J. Hum. Genet..

[5] Hong W., Haviland I., Pestana-Knight E., Weisenberg J.L., Demarest S., Marsh E.D., Olson H.E. (2022). CDKL5 Deficiency Disorder-Related Epilepsy: A Review of Current and Emerging Treatment. CNS Drugs.

[6] Olson H.E., Demarest S.T., Pestana-Knight E.M., Swanson L.C., Iqbal S., Lal D., Leonard H., Cross J.H., Devinsky O., Benke T.A. (2019). Cyclin-Dependent Kinase-Like 5 Deficiency Disorder: Clinical Review. Pediatr. Neurol..

[7] Jakimiec M., Paprocka J., Śmigiel R. (2020). CDKL5 Deficiency Disorder-A Complex Epileptic Encephalopathy. Brain Sci..

[8] Bahi-Buisson N., Nectoux J., Rosas-Vargas H., Milh M., Boddaert N., Girard B., Cances C., Ville D., Afenjar A., Rio M. (2008). Key Clinical Features to Identify Girls with CDKL5 Mutations. Brain.

[9] Van Bergen N.J.V., Massey S., Quigley A., Rollo B., Harris A.R., Kapsa R.M.I., Christodoulou J. (2022). CDKL5 Deficiency Disorder: Molecular Insights and Mechanisms of Pathogenicity to Fast-Track Therapeutic Development. Biochem. Soc. Trans..

[10] Rusconi L., Salvatoni L., Giudici L., Bertani I., Kilstrup-Nielsen C., Broccoli V., Landsberger N. (2008). CDKL5 Expression is Modulated During Neuronal Development and its Subcellular Distribution is Tightly Regulated by the C-terminal Tail. J. Biol. Chem..

[11] Fuchs C., Trazzi S., Torricella R., Viggiano R., De Franceschi M., Amendola E., Gross C., Calzà L., Bartesaghi R., Ciani E. (2014). Loss of CDKL5 Impairs Survival and Dendritic Growth of Newborn Neurons by Altering AKT/GSK-3β Signaling. Neurobiol. Dis..

[12] Della Sala G., Putignano E., Chelini G., Melani R., Calcagno E., Ratto G.M., Amendola E., Gross C.T., Giustetto M., Pizzorusso T. (2016). Dendritic Spine Instability in a Mouse Model of CDKL5 Disorder Is Rescued by Insulin-like Growth Factor 1. Biol. Psychiatry.

[13] Wang I.T., Allen M., Goffin D., Zhu X., Fairless A.H., Brodkin E.S., Siegel S.J., Marsh E.D., Blendy J.A., Zhou Z. (2012). Loss of CDKL5 Disrupts Kinome Profile and Event-Related Potentials Leading to Autistic-Like Phenotypes in Mice. Proc. Natl. Acad. Sci. USA.

[14] Bahi-Buisson N., Bienvenu T. (2012). CDKL5-Related Disorders: From Clinical Description to Molecular Genetics. Mol. Syndromol..

[15] Hector R.D., Kalscheuer V.M., Hennig F., Leonard H., Downs J., Clarke A., Benke T.A., Armstrong J., Pineda M., Bailey M.E.S. (2017). CDKL5 Variants: Improving our Understanding of a Rare Neurologic Disorder. Neurol. Genet..

[16] Szafranski P., Golla S., Jin W., Fang P., Hixson P., Matalon R., Kinney D., Bock H.G., Craigen W., Smith J.L. (2015). Neurodevelopmental and Neurobehavioral Characteristics in Males and Females with CDKL5 Duplications. Eur. J. Hum. Genet..

[17] Kalscheuer V.M., Tao J., Donnelly A., Hollway G., Schwinger E., Kübart S., Menzel C., Hoeltzenbein M., Tommerup N., Eyre H. (2003). Disruption of the Serine/Threonine Kinase 9 Gene Causes Severe X-Linked Infantile Spasms and Mental Retardation. Am. J. Hum. Genet..

[18] Nishimura A., Takano T., Mizuguchi T., Saitsu H., Takeuchi Y., Matsumoto N. (2008). CDKL5 Disruption by t(X;18) in a Girl with West Syndrome. Clin. Genet..

[19] Cope H., Barseghyan H., Bhattacharya S., Fu Y., Hoppman N., Marcou C., Walley N., Rehder C., Deak K., Alkelai A. (2021). Detection of a Mosaic CDKL5 Deletion and Inversion by Optical Genome Mapping ends an Exhaustive Diagnostic Odyssey. Mol. Genet. Genomic Med..

[20] Darra F., Monchelato M., Loos M., Juanes M., Bernardina B.D., Valenzuela G.R., Gallo A., Caraballo R. (2023). CDKL5-associated developmental and epileptic encephalopathy: A long-term, longitudinal electroclinical study of 22 cases. Epilepsy Res..

[21] McKinlay Gardner R.J., Amor D.J. (2018). Inversions. Gardner and Sutherland’s Chromosome Abnormalities and Genetic Counselling.

[22] Schluth-Bolard C., Diguet F., Chatron N., Rollat-Farnier P.A., Bardel C., Afenjar A., Amblard F., Amiel J., Blesson S., Callier P. (2019). Whole Genome Paired-End Sequencing Elucidates Functional and Phenotypic Consequences of Balanced Chromosomal Rearrangement in Patients with Developmental Disorders. J. Med. Genet..

[23] Xin Y., Zhou J., Ding Q., Chen C., Wu X., Wang X., Wang H., Jiang X. (2017). A Pericentric Inversion of Chromosome X Disrupting F8 and Resulting in Haemophilia A. J. Clin. Pathol..

[24] Halgren C., Nielsen N.M., Nazaryan-Petersen L., Silahtaroglu A., Collins R.L., Lowther C., Kjaergaard S., Frisch M., Kirchhoff M., Brøndum-Nielsen K. (2018). Risks and Recommendations in Prenatally Detected De Novo Balanced Chromosomal Rearrangements from Assessment of Long-Term Outcomes. Am. J. Hum. Genet..

[25] Sahajpal N.S., Barseghyan H., Kolhe R., Hastie A., Chaubey A. (2021). Optical Genome Mapping as a Next-Generation Cytogenomic Tool for Detection of Structural and Copy Number Variations for Prenatal Genomic Analyses. Genes.

[26] Schnause A.C., Komlosi K., Herr B., Neesen J., Dremsek P., Schwarz T., Tzschach A., Jägle S., Lausch E., Fischer J. (2021). Marfan Syndrome Caused by Disruption of the FBN1 Gene due to A Reciprocal Chromosome Translocation. Genes.

[27] Fang X., Butler K.M., Abidi F., Gass J., Beisang A., Feyma T., Ryther R.C., Standridge S., Heydemann P., Jones M. (2022). Analysis of X-Inactivation Status in a Rett Syndrome Natural History Study Cohort. Mol. Genet. Genomic Med..

[28] Fehr S., Leonard H., Ho G., Williams S., de Klerk N., Forbes D., Christodoulou J., Downs J. (2015). There is Variability in the Attainment of Developmental Milestones in the CDKL5 Disorder. J. Neurodev. Disord..

[29] Masliah-Plachon J., Auvin S., Nectoux J., Fichou Y., Chelly J., Bienvenu T. (2010). Somatic Mosaicism for a CDKL5 Mutation as an Epileptic Encephalopathy in Males. Am. J. Med. Genet. A.

[30] Kato T., Morisada N., Nagase H., Nishiyama M., Toyoshima D., Nakagawa T., Maruyama A., Fu X.J., Nozu K., Wada H. (2015). Somatic Mosaicism of a CDKL5 Mutation Identified by Next-Generation Sequencing. Brain Dev..

[31] Liang J.S., Huang H., Wang J.S., Lu J.F. (2019). Phenotypic Manifestations between Male and Female Children with CDKL5 Mutations. Brain Dev..

[32] Rodak M., Jonderko M., Rozwadowska P., Machnikowska-Sokołowska M., Paprocka J. (2022). CDKL5 Deficiency Disorder (CDD)-Rare Presentation in Male. Children.

[33] Sini V., Kamga K.A.W., Bimbaï A.M., Kamga O.J.P. (2021). Psychopathologic Disorders Associated with Epilepsy at the Yaoundé General Hospital. Afr. J. Neurol. Sci..

[34] Siri B., Varesio C., Freri E., Darra F., Gana S., Mei D., Porta F., Fontana E., Galati G., Solazzi R. (2021). CDKL5 Deficiency Disorder in Males: Five New Variants and Review of the Literature. Eur. J. Paediatr. Neurol..

[35] Mirzaa G.M., Paciorkowski A.R., Marsh E.D., Berry-Kravis E.M., Medne L., Alkhateeb A., Grix A., Wirrell E.C., Powell B.R., Nickels K.C. (2013). CDKL5 and ARX Mutations in Males with Early-Onset Epilepsy. Pediatr. Neurol..

[36] Akabori S., Takano T., Fujito H., Takeuchi Y. (2007). West Syndrome in a Patient with Balanced Translocation t(X;18)(p22;p11.2). Pediatr. Neurol..

[37] Allou L., Julia S., Amsallem D., El Chehadeh S., Lambert L., Thevenon J., Duffourd Y., Saunier A., Bouquet P., Pere S. (2017). Rett-Like Phenotypes: Expanding the Genetic Heterogeneity to the KCNA2 Gene and First Familial Case of CDKL5-Related Disease. Clin. Genet..

[38] Kopanos C., Tsiolkas V., Kouris A., Chapple C.E., Albarca Aguilera M., Meyer R., Massouras A. (2019). VarSome: The Human Genomic Variant Search Engine. Bioinformatics.

[39] Richards S., Aziz N., Bale S., Bick D., Das S., Gastier-Foster J., Grody W.W., Hegde M., Lyon E., Spector E. (2015). Standards and Guidelines for the Interpretation of Sequence Variants: A Joint Consensus Recommendation of the American College of Medical Genetics and Genomics and the Association for Molecular Pathology. Genet. Med..

[40] Robinson J.T., Thorvaldsdóttir H., Winckler W., Guttman M., Lander E.S., Getz G., Mesirov J.P. (2011). Integrative, Genomics Viewer. Nat. Biotechnol..

[41] Busque L., Paquette Y., Provost S., Roy D.C., Levine R.L., Mollica L., Gilliland D.G. (2009). Skewing of X-inactivation Ratios in Blood Cells of Aging Women is Confirmed by Independent Methodologies. Blood.

[42] Sharp A., Robinson D., Jacobs P. (2000). Age-and Tissue-Specific Variation of X Chromosome Inactivation Ratios in Normal Women. Hum. Genet..

[43] Allen R.C., Zoghbi H.Y., Moseley A.B., Rosenblatt H.M., Belmont J.W. (1992). Methylation of HpaII and HhaI Sites Near the Polymorphic CAG Repeat in the Human Androgen-Receptor Gene Correlates with X Chromosome Inactivation. Am. J. Hum. Genet..

[44] Amos-Landgraf J.M., Cottle A., Plenge R.M., Friez M., Schwartz C.E., Longshore J., Willard H.F. (2006). X Chromosome-Inactivation Patterns of 1,005 Phenotypically Unaffected Females. Am. J. Hum. Genet..

[45] Giorda R., Bonaglia M.C., Beri S., Fichera M., Novara F., Magini P., Urquhart J., Sharkey F.H., Zucca C., Grasso R. (2009). Complex Segmental Duplications Mediate a Recurrent dup(X)(p11.22-p11.23) Associated with Mental Retardation, Speech Delay, and EEG Anomalies in Males and Females. Am. J. Hum. Genet..

